# Pollinator individual‐based networks reveal the specialized plant–pollinator mutualism in two biodiverse communities

**DOI:** 10.1002/ece3.8384

**Published:** 2021-11-25

**Authors:** Lin‐Lin Wang, Yong‐Ping Yang, Yuan‐Wen Duan

**Affiliations:** ^1^ Germplasm Bank of Wild Species Kunming Institute of Botany Chinese Academy of Sciences Kunming China; ^2^ University of Chinese Academy of Sciences Beijing China; ^3^ Institute of Tibetan Plateau Research at Kunming Kunming Institute of Botany Chinese Academy of Sciences Kunming China

**Keywords:** generalization, individual‐based network, pollination network, Qinghai–Tibet Plateau, specialization

## Abstract

Generalization of pollination systems is widely accepted by ecologists in the studies of plant–pollinator interaction networks at the community level, but the degree of generalization of pollination networks remains largely unknown at the individual pollinator level. Using potential legitimate pollinators that were constantly visiting flowers in two alpine meadow communities, we analyzed the differences in the pollination network structure between the pollinator individual level and species level. The results showed that compared to the pollinator species‐based networks, the linkage density, interaction diversity, interaction evenness, the average plant linkage level, and interaction diversity increased, but connectance, degree of nestedness, the average of pollinator linkage level, and interaction diversity decreased in the pollinator individual‐based networks, indicating that pollinator individuals had a narrower food niche than their counterpart species. Pollination networks at the pollinator individual level were more specialized at the network level (*H*′_2_) and the plant species node level (*d*′) than at the pollinator species‐level networks, reducing the chance of underestimating levels of specialization in pollination systems. The results emphasize that research into pollinator individual‐based pollination networks will improve our understanding of the pollination networks at the pollinator species level and the coevolution of flowering plants and pollinators.

## Graphical abstract

Our finding revealed that the pollinator individual‐level pollination networks were specialized at network and plant node level. The pollinator individual‐based pollination networks reduce the chance of underestimating levels of specialization and improve our understanding of coevolution of flowering plants and pollinators in pollination systems.

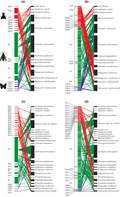

## 1 INTRODUCTION

The interactions between plants and their pollinators are considered to be one of the fundamental drivers of angiosperm diversity (Stebbins, 1970). The specialization of the pollination system is important for a successful pollination, since a high level of specialization would assure conspecific pollen transfers within plant species and a high energy intake rate of pollinators (Johnson & Steiner, 2000). Local populations of entomophilous plant species would benefit from specializing foraging bouts of pollinator individuals in a short time with no temporal changes caused by adjacent factors. Reducing the interspecific pollen transfer would ensure seed production via outcrossing and species integrity by reducing pollen flows among multiple species (Morales & Traveset, 2008; Waser, 1978). Specialization of the pollination system could improve individual pollinators’ energy intake rates by reducing the energy costs of learning new foraging behaviors in adapting new flower structures during shifts to new plant species (Chittka et al., 1999; Waser, 1986). Although the specialized pollination systems are of ecological advantage for both plant species and pollinator individuals (Armbruster, 2017), the number of pollinator individual‐based interaction network studies on a community‐wide level has so far been very scarce.

Studies of plant–pollinator interaction networks play an important role in understanding the generalization and specialization of pollination systems (Bascompte et al., 2003; Olesen et al., 2007). The generalized character of pollination networks has been widely accepted (Blüethgen et al., 2007); however, most studies on pollination networks lack biological details that describe flower‐visiting insects as pollinators (de Santiago‐Hernández et al., 2019; Guimarães, 2020). This is acceptable for food webs, but inappropriate when assessing generalized versus specialized pollination in communities. In addition, most pollination networks are constructed based on flower visitor species (Bascompte & Jordano, 2007; Memmott, 1999), but these species‐based pollination networks may not reflect the true pollination interactions between plants and their pollinators (de Santiago‐Hernández et al., 2019; Guimarães, 2020). Indeed, in traditional species‐based pollination networks, pollinator species are aggregates of pollinator individuals, which are the true pollination links observed in nature. In addition, pollinator species‐level interaction networks are aggregates of different plants and pollinators species over a long period, which may obscure the pollinator individual behavior and increase forbidden links between interacting mutualists (Ings et al., 2009; Olesen et al., 2011). In this context, plant–pollinator relationships at the community levels could be more accurately represented by combining pollinator individual‐based networks with pollinator’ foraging behavior.

**FIGURE 1:**
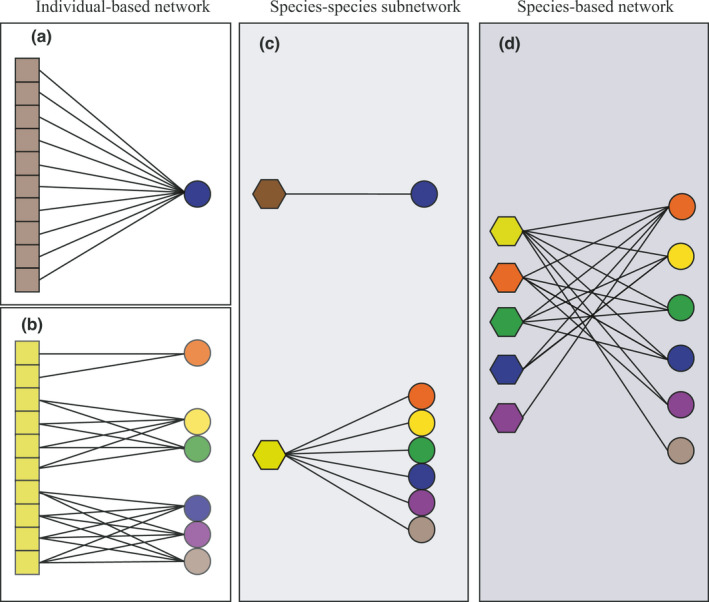
Pollinator individual‐based network and species‐based network. At the pollinator population level, different pollinator individuals (squares) can interact with one (a) or lots of flowering plant species (b) (circles; different colors represent different flowering plant species), leading to individual‐based networks within pollinator populations. At the pollinator species level, these individual‐based networks result in a species‐species subnetwork (c, hexagons; different colors represent different pollinator species). Finally, at the community level, the species–species subnetworks combine with each other to form species‐based networks (d)

As species are assemblages of individuals, pollination networks are organized hierarchically and can be scaled down from species‐based pollination networks to individual‐based ones (Dupont et al., 2011, 2014; Lucas et al., 2018; Olesen et al., 2010; Tur et al., 2014). Then, pollination networks can be built at two levels of resolution: pollinator individual‐plant species network and pollinator species‐plant species network (Figure 1). Individuals–species networks represent interactions between pollinator individuals and plant species (Figure 1a,b), and species–species networks represent interactions between pollinator species and plant species (Figure 1c,d). However, to date, few empirical studies have attempted to explore the interaction networks between flowering plant species and pollinators at the individual level. For example, the network of the individual thistles *Cirsium arvense* and the honeybee *Apis mellifera* was more closely linked than previous knowledge of species pollination networks indicated (Dupont et al., 2011). Tur et al. (2014) suggested that individual flower visitor‐plant species pollen transport networks were more specialized than species–species networks, and generalist pollinator species often comprised specialist pollinator individuals. The two limited cases strongly suggest that pollination networks based on pollinator individual level could be more specialized than those based on pollinator species level (Araújo et al., 2011; Des Roches et al., 2018; Tonos et al., 2021), indicating that pollination networks based on pollinator individual level could fill the gap between high pollinator fidelity and the highly generalized pollination networks (Arceo‐Gómez et al., 2016; Lázaro et al., 2008).

It is widely believed that the relationships between plants and pollinators are highly generalized because most pollinator species visit many plant species across broad time scales at the community levels and vice versa (Waser et al., 1996). This has been strongly supported by community studies on pollination networks over the past two decades (Blüethgen et al., 2007; Memmott, 1999; Petanidou et al., 2008). Building pollination networks based on flower visitors that contact the reproductive organs (Kaiser‐Bunbury et al., 2017; Memmott, 1999), identification of pollen carried by flower visitors (Lucas et al., 2018; Tur et al., 2014), pollen on stigmas (Fang & Huang, 2013), and the direct assessment of pollinator effectiveness (de Santiago‐Hernández et al., 2019; Traveset et al., 2015) can provide accurate assessments of the specialization of pollination networks. Therefore, in the present study, we structured pollination networks at the pollinator individual level by tracking the foraging behaviors of pollinators among plants, together with pollination networks at species level (Figure 1), in two alpine meadow communities. Specifically, we addressed the following specific questions: (1) What were the differences between the structures of pollinator networks at the pollinator individual‐level and species‐level? (2) How did the level of specialization of pollinator individual‐based networks change in comparison with pollinator species‐based networks? We expected that the linkage density, interaction diversity, interaction evenness, the average plant linkage level, and interaction diversity would increase, but connectance, degree of nestedness, the average pollinator linkage level, and interaction diversity in the pollinator individual‐based networks would decrease due to individual pollinator specialization and the increased number of pollinator nodes when downsizing from pollinator species‐based networks to individual‐based networks (Brosi, 2016; Tur et al., 2014). We also expected that pollinator individual‐based networks would be a higher level of specialization than pollinator species‐based networks because most pollinator individuals may show a high degree of specialization and floral fidelity over individual foraging bouts (Araújo et al., 2011; Arroyo‐Correa et al., 2021; Brosi, 2016).

## 2 MATERIALS AND METHODS

### 2.1 Experimental sites

This study was conducted in 2019 at two alpine communities on the northeastern Qinghai–Tibet Plateau in Menyuan county (3210 m above sea level, 101°12′E, 37°29′N) and Huangyuan county (3120 m above sea level, 101°16′E, 36°31′N), Qinghai Province, China. The two sites of are c. 100 km apart from each other (Appendix 1). The two alpine communities are characterized by long winters and short summers. The mean annual temperature and precipitation were 3°C and 410 mm, respectively. Approximately 80% of the precipitation falls during the growing seasons from June to September. For pollinator observations, we built a large, fenced plot (100 m * 100 m) in each of the two communities in June 2019. We chose the two large plots in natural alpine communities with similar environmental conditions and vegetation. In the two plots, the principal plant groups are graminoids and forbs. The plots consisted of more than 50 flowering plant species (most of them from Fabaceae, Asteraceae, Ranunculaceae, Rosaceae, and Gentianaceae) that were found in nearby alpine meadows. Among the flowering plants, *Potentilla* spp., *Oxytropis* spp., *Pedicularis* spp., *Cirsium carvi*, and *Halenia elliptica* are the most abundant. The dominant pollinator species are Diptera and Hymenoptera (such as *Apis mellifera*, *Bombus supremus*, and *B. asiaticus*).

### 2.2 Tracking pollinators

Because effective pollen dispersal distance on our alpine meadows was less than 8 m (Wang et al., 2021), we built four small plots (10 m * 10 m) in the large plots (100 m *100 m) to observe the foraging behavior of pollinator (Appendix 1). We chose the same 4 small plots (10 m *10 m) to observe all the flowering plants in the large plots as much as possible. Quantifying the movements of pollinators was practical in relatively small quadrat, as pollinators tend to forage on nearby plant species (Fang & Huang, 2016; Waser, 1986). Pollinator visitations were observed from late June to late August 2019 within the peak season of pollinator activity. The distance between the flowering plants was no more than 4 m, within the distance at which we could follow pollinator movements.

To track the movements of the pollinators, four fixed 1 m * 1 m observation quadrats were set up in the middle of small plots (Appendix 1, 10 m * 10 m). Each quadrat had 5–8 (Mean ± SD = 6.4 ± 0.9) flowering plant species. We selected one fixed quadrat and waited for potential pollinators to visit the open flowers. When a flower‐visiting insect entered the selected fixed quadrat (1 m * 1 m) and visited the flowers, we tracked the insect as far as possible, and until it left the selected small plot (10 m * 10 m). The insect species, plant species, and the number of visited flowers were recorded. Each quadrat (1 m * 1 m) was monitored every half month for 30 min per session, for four sessions on different days. Following Memmott (1999), we considered a visitor to be a potential pollinator if it came into contact with the reproductive structure (anthers and stigmas) of flowers while actively searching for pollen or nectar. We only recorded a flower‐visiting insect that came into contact with anthers and stigmas of at least two flowers, because effective pollinator‐mediated pollination mainly occurs between different flowers (Armbruster, 2017). A floral unit was defined as one or many flowers (such as the flower heads of Umbelliferae and Asteraceae; Fang & Huang, 2016). We tracked flower‐visiting insects between 0900 and 1900 hours on clear days with no strong wind. We did not perform any observations outside of this period and conditions as pollinator activities are limited due to the low temperature at high altitudes (Fang & Huang, 2016). All pollinators were insects in the orders: Diptera, Hymenoptera, and Lepidoptera. We collected all the pollinators that continuously visited flowers when they left the selected plot, and identified them with the help of taxonomist experts. We observed a total of 28 flowering species (Appendix 2) over 64 h (0.5 h *4 sessions *4 quadrats * 4 small plots * 2 large plots), which were visited by 43 pollinator species (Appendix 3).

### 2.3 Statistical analysis

To compare the differences between pollinator individual‐ and species‐based networks, we first constructed pollination networks at two levels of resolution: pollinator individual‐plant species network and pollinator species‐plant species network (Figure 1). The pollinator individual‐based networks were constructed using observed pollinator individuals and plant species as nodes. The pollinator species‐based networks were constructed using observed pollinators and plant species as nodes. Interaction weight was defined as the number of flowers visited by either pollinator individuals or species.

To analyze differences between pollinator individual‐ and species‐level networks, we selected the following parameters to describe the pollination network structure: number of pollinator nodes, number of plant nodes, total number of nodes, total number of interactions, linkage level (number of interactions of each network node), network size (total number of possible interactions in the network), linkage density (mean number of links per network node), connectance (realized proportion of all possible links), interaction diversity (Shannon diversity of links for the whole network), interaction evenness (Shannon's evenness of link frequency distribution in the whole network), and nestedness (NODF: what extent the interaction pattern resembles a perfectly nested pattern). We use the constrained model with fixed row and column sums (linkage level was fixed) to assess the significance of the NODF metric (1000 randomizations). In addition, we use a one‐tailed *Z* test to quantify the possibility of randomly getting higher NODF values than the experimental matrix networks.

To examine whether the network structure changes due to the change in network size when switching from the pollinator species‐based networks to the pollinator individual‐based networks, we constructed 1000 null networks that have the same species composition and network size as the empirical pollinator individual‐based networks. These null networks were built by combining simulated pollinator individual‐based sub‐matrices for each species generated using the Patefield algorithm (i.e., observed marginal sums maintained for rows and columns of the matrix). Each null pollinator individual‐based subnetwork simulated that the related individuals act as generalists as their species, sampling each plant species at a rate proportional to the corresponding species visitation distribution. Thus, in null pollinator individual‐based sub‐matrices with X rows and Y columns. X was the number of individuals of species A, and Y was the number of plant species visited by species A. Each individual was reassigned the same visits as observed, but visits were randomly distributed among plant species with a probability equal to the observed plant species proportion. The abovementioned parameters were calculated for the 1000 null model networks. When parameter values of empirical pollinator individual‐based networks did not fall into 95% confidence intervals of values for the null networks, differences were thus attributed to individual specialization and not to the change in network size. We also evaluate the network specialization (*H*′_2_), mean pollinator specialization (*d*′*p_oll_*), and mean plant specialization (*d*′*p_l_*). Independent *t* tests were used to compare the specialization of nodes between pollinator species‐based and individual‐based networks at Menyuan and Huangyuan sites, respectively. All network metrics were implemented with the bipartite (version 2.15) and vegan (version 2.5‐7) packages in the R statistical software version 3.5.3 (R Development Core Team, 2019).

## 3 RESULTS

**FIGURE 2:**
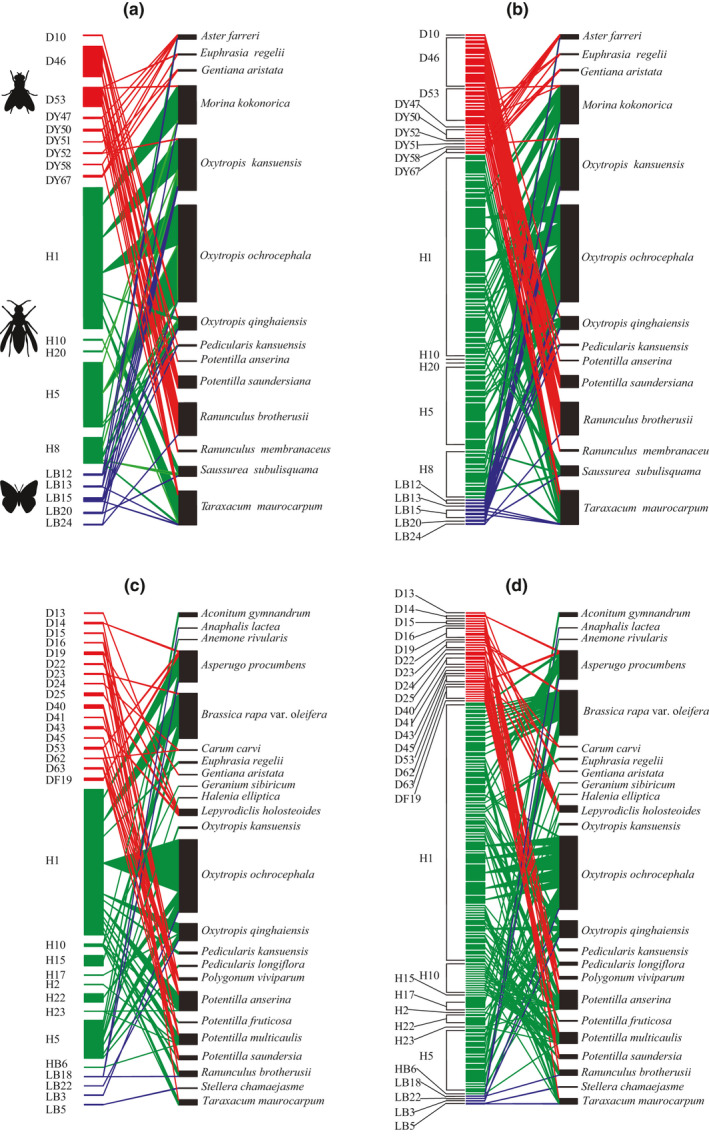
Pollination networks at two levels of resolution. Pollination networks of pollinator species‐level (a, c) and individual‐level (b, d) at Menyuan (left) and Huangyuan (right), on the Qinghai–Tibet Plateau, China. The networks depict bipartite quantitative networks of interactions (links) between flowering plant species (right bars) and pollinator individuals or species (left bars). Each block represents a species or individual. The width of a block reflects the relative abundance of flowers and pollinators. Color triangles are pollination interactions between plants and pollinators, and the width of the links shows the interaction number between pollinators and plants. Bar width is proportional to the number of interactions. Colors depict pollinator groups: red, flies; green, wasps and bees; blue, moths and butterflies

In two alpine meadows, we tracked 208 pollinator individuals, which visited at least two flowers. Hymenoptera, Diptera, and Lepidoptera consecutively visited 51.2 ± 70.9 (*n* = 133), 14.6 ± 16.7 (*n* = 62), and 7.1 ± 5.6 (*n* = 13) (mean ± SD) flowers, and accounted for 87.3%, 11.6%, and 1.1%, respectively, of 7839 visits. In the Menyuan plot, a total of 84 pollinator individuals (Hymenoptera: 48, Diptera: 28, and Lepidoptera: 8), from 19 pollinator species (Hymenoptera: 5, Diptera: 9, and Lepidoptera: 5), visited 14 flowering plants (Table 1; Figure 2a,c). In the Huangyuan plot, a total of 124 pollinator individuals (Hymenoptera: 85, Diptera: 34, and Lepidoptera: 5), from 30 pollinator species (Hymenoptera: 9, Diptera: 17, and Lepidoptera: 4), visited 24 flowering plants (Table 1; Figure 2b,d).

**TABLE 1:** Parameters of pollinator species‐ and individual‐level pollination networks at Menyuan and Huangyuan plots, on the Qinghai–Tibet Plateau, China

Parameters	Menyuan			Huangyuan		
	Species	Individual	Null model	Species	Individual	Null model
Number of pollinator nodes	19	84	84	30	124	124
Number of plant nodes	14	14	14	24	24	24
Total number of nodes	33	98	98	54	148	148
Total number of interactions	63	136^†^	285.86	69	150^†^	704.34
Linkage density	2.95	4.84^†^	2.89	3.86	4.42^†^	4.24
Interaction diversity	2.78	4.04^†^	4.96	2.96	4.12^†^	5.60
Interaction evenness	0.50	0.57^†^	0.70	0.45	0.52^†^	0.69
Connectance	0.24	0.12^†^	0.24	0.10	0.05^†^	0.21
Nestedness (NODF)	38.32 ^ns^	14.37*^,^ ^†^	36.81	19.45*	4.46*^,^ ^†^	51.18
Mean pollinator linkage level	3.32	1.62^†^	3.38	2.30	1.21^†^	4.96
Mean pollinator interaction diversity	0.78	0.27^†^	0.90	0.38	0.09^†^	1.08
Mean plant linkage level	4.50	9.17^†^	3.37	2.88	6.25^†^	4.96
Mean plant interaction diversity	0.79	1.67^†^	0.90	0.40	1.00^†^	1.08

^†^ Observed values were outside of 95% confidence intervals of values obtained for 1000 null pollinator individual‐based networks. NS: significance *p*‐value > .05; *p‐value < .05. That is the probability of getting by random a higher value of nestedness than the empirical one.

Most of the topological network parameters examined changed due to downsizing from pollinator species‐based networks to pollinator individual‐based networks (Table 1). Empirical pollinator individual‐based networks appeared to be larger than pollinator species‐based networks in network size, because 42% of pollinator nodes consisted of multiple individuals (Figure 2). As a result, the downsizing at both study sites increased the total number of interactions by about 2.1 times (Table 1; Figure 2), although considerably less than expected with null pollinator individual‐based networks (Table 1). Network connectances at both Menyuan and Huangyuan sites in empirical pollinator individual‐based networks were less than half compared with the null hypothesis (Table 1). Furthermore, interaction diversity and interaction evenness in empirical pollinator individual‐based networks were significantly reduced compared with the null models due to the differences in the number of interactions (Table 1). Therefore, such differences between pollinator species‐based and individual‐based networks can be attributed to a significant decrease in the average number of links of pollinator nodes in empirical pollinator individual‐based networks (Table 1), rather than to an impact of increasing network size. Both pollinator species‐based and individual‐based networks at two study sites were insignificantly nested, except the pollinator species‐based network at Menyuan site. However, the NODF values were significantly lower in empirical pollinator individual‐based networks than in null model networks (Table 1). The mean degree pollinator linkage level in pollinator individual‐based networks was about 50% lower than that predicted by the null model. The mean interaction diversity for pollinators was significantly less when downsizing from pollinator species‐based networks to individual‐based networks, suggesting that pollinator individuals had a narrower food niche than their counterparts (Table 1).

In both study sites, the network specialization metrics (*H*′_2_) showed that the pollinator individual‐based networks were highly specialized, with values of the network specializations at two sites were greater than 0.9 (Table 2). Downsizing from pollinator species‐based networks to individual‐based networks increased the network specializations (*H*′_2_) 1.7‐fold and 2.1‐fold at Menyuan and Huangyuan sites, respectively (Table 2). Mean plant specialization (*d*′*p_l_*) of the pollinator individual‐based networks was higher than that of the pollinator species‐based networks at both sites (Table 2). The mean value of *d*′ for all plant species was 0.85, indicating that most plant species were the unique interactions in the pollinator individual‐based networks. In addition, the mean value of *d*′ for all pollinators was 0.44, indicating that about half of the interactions were unique for pollinator individuals (Table 2).

**TABLE 2:** Values of network specialization (H′2) and the specialization of pollinator (d′poll) and plant nodes (d′plant) at two alpine grassland sites (Menyuan and Huangyuan) in Qinghai, China

Parameters	Menyuan		Huangyuan	
	Species	individuals	Species	individuals
Specialization of networks (*H*′_2_)	0.54	0.91	0.47	0.98
Specialization of pollinator nodes (*d*′*p_oll_*)	0.39 ± 0.19a	0.42 ± 0.15a	0.42 ± 0.23a	0.50 ± 0.19a
Specialization of plant nodes (*d'p_l_*)	0.41 ± 0.23a	0.79 ± 0.19b	0.42 ± 0.33a	0.88 ± 0.18b

Note

Independent *t* tests were used to compare the specialization of nodes between pollinator species‐based and individual‐based networks at Menyuan and Huangyuan sites, respectively. Different letters indicate significant differences at .05 level.

## 4 DISCUSSION

In this study, using potential legitimate pollinators who continuously visited flowers (Fang & Huang, 2016; Freitas, 2013), we explored the specialization of the pollinator individual‐level and species‐level pollination networks in two alpine meadow communities. Our results demonstrate that the pollinator individual‐level pollination networks are specialized at network and node levels. This study shows the importance of considering the value of individual pollinators to examine the plant–pollinator interaction networks at the community level.

Specialized pollination systems are necessary for flowering plants as well as pollinators because most flowering plants need pollinators to transfer conspecific pollen between individuals to produce seed (Brosi, 2016; Darwin, 1876), and to ensure high foraging rates, most pollinator individuals restrict their visits to flowers of a single species or species morph (Chittka et al., 1999; Waser, 1986). In the current study, the values of specialization level at the whole pollinator individual‐based networks are larger than 0.9 (0.91 at Menyuan; 0.98 at Huangyuan sites). These values are higher than those recorded in all pollinator species‐level pollination networks in the web of life (https://www.web‐of‐life.es). A possible explanation might be that a single pollinator in pollinator individual‐based networks shows a high degree of specialization due to floral fidelity over the short term (Brosi, 2016; Brosi & Briggs, 2013). Our results showed that most pollinator individuals had a high degree of floral fidelity over pollinator individual foraging bouts in the alpine grassland. This finding was consistent with that of Tur et al. (2014), who found that network downscaling indicated high specialization of pollinator individuals. However, both of the two pollinator species‐based networks in our study sites were moderately specialized (0.54 at Menyuan; 0.47 at Huangyuan sites), which are comparable to the degree of specialization (from 0.24 to 0.85) in previous pollination network researches over the long term (Blüethgen et al., 2007). Our results support the view that pollination systems might be specialized in the short term to ensure conspecific pollen transfer but generalized over the long term to ensure the robustness of pollination system (Brosi, 2016). Moreover, our results also showed that the mean value of *d*′ for all plant species was 0.85, indicating that the most pollinator individuals visited a few numbers of plant species in the alpine communities (Figure 2), ensuring effective pollen transfer within the species (Fang & Huang, 2016). It is worth noting that our research only focused on large flying insects (i.e., bees, flies, and butterflies), but some small insects with poor mobility (ants, beetles, thrips, etc.) can also provide pollination services for some alpine plants that are not easily visited by large insects. Future research needs to pay attention to the pollination ability of these insects at the community level.

All pollination network studies combined data on a specific time scale. For example, numerous pollination networks are constructed from highly aggregated information from daily field samples to weeks or entire seasons (Olesen et al., 2010; Petanidou et al., 2008; Schwarz et al., 2020). The structure of pollination networks could change significantly from 1 day to many years due to the temporal dynamics of species diversity, species turnover, and the rewiring of interaction over time scales (CaraDonna et al., 2017; Petanidou et al., 2008; Souza et al., 2018). In our study, we collected 2 months of data on alpine meadows in order to structure the pollination network, which may encompass some species with non‐overlapping phenology and introduce temporally forbidden links into the networks. For example, a pollination network only contains species that are simultaneously active at the same time if the pollination network was established over a single day or week. The pollination network across the entire growing season, however, encompasses both early, medium, and late flowering plants as well as pollinators that only occur at certain times within the season. A high temporal variation of species and the rewiring of links over the entire growing season can consequently prevent the establishment of a highly connected network core and thus limit the specialization of the entire network and nodes (Schwarz et al., 2020; Seifert et al., 2021). For example, species can appear as specialists in networks that are combined over narrow time scales, such as 1 day or week. In contrast, they can appear as generalists in networks that are combined over broad time scales, such as a survey season or year if they change interaction partners throughout the season (Chávez‐González et al., 2020; Olesen et al., 2008; Vitt et al., 2020). Therefore, further research is required to examine the structure of pollinator individual‐based pollination networks across a variety of time scales and to fully understand the implications of the pollination network structure.

In addition, most studies on pollination networks cannot assess whether flower‐visiting insects have come into contact with anthers and stigmas from at least two flowers, since effective pollinator‐mediated pollination occurs mainly between different flowers, especially self‐incompatible plants (Armbruster, 2017), which can cause misinterpretation of the degree of pollinator specialization (Armbruster, 2017; Brosi, 2016). In our study, the main pollinator species are flies and bees, and these insects only feed on or collect pollen and nectar (personal observations). However, some flower visitors do not continuously visit flowers, indicating that these flower visitors did not transfer conspecific pollen (Ballantyne et al., 2015; de Santiago‐Hernández et al., 2019; Wang et al., 2017), which could result in an underestimation of pollinator specialization (de Santiago‐Hernández et al., 2019). For example, when a pollinator visits the flowers of one or few plant species for pollen and nectar over individual foraging bouts, it would normally be treated as a specialist. However, different pollinator individuals need several species of floral resources from their environment—these may have to be harvested from a variety of flowers, and the pollinator species may be treated as a generalist. Therefore, in future research, pollination networks need to pay more attention to effective pollen transfer by different pollinator individuals, to reflect the specialization of the interaction network between plants and pollinators.

## CONFLICT OF INTEREST

The authors declare no competing interest.

## AUTHOR CONTRIBUTION

**Lin‐Lin Wang:** Conceptualization (equal); Funding acquisition (equal); Investigation (equal); Methodology (equal); Software (equal); Visualization (equal); Writing‐original draft (equal). **Yongping Yang:** Conceptualization (equal); Funding acquisition (equal); Supervision (equal); Writing‐original draft (equal); Writing‐review & editing (equal). **Yuan Wen Duan:** Conceptualization (equal); Funding acquisition (equal); Software (equal); Supervision (equal); Visualization (equal); Writing‐original draft (equal); Writing‐review & editing (equal).

## Data Availability

Data deposited in the Dryad repository: https://doi.org/10.5061/dryad.cz8w9gj3j.

## APPENDIX 2

Plant species list visited continuously by pollinators. √ indicates flowering plants existing at Menyuan or Huangyuan plots

**Table:**

Plant species	Menyuan	Huangyuan
*Aconitum gymnandrum*		√
*Anaphalis lactea*		√
*Anemone rivularis*		√
*Asperugo procumbens*		√
*Aster farreri*	√	
*Brassica rapa* var. *oleifera*		√
*Carum carvi*		√
*Dasiphora fruticosa*		√
*Euphrasia regelii*	√	√
*Gentiana aristata*	√	√
*Geranium sibiricum*		√
*Halenia elliptica*		√
*Lepyrodiclis holosteoides*		√
*Morina kokonorica*	√	
*Oxytropis kansuensis*	√	√
*Oxytropis ochrocephala*	√	√
*Oxytropis qinghaiensis*	√	√
*Pedicularis kansuensis*	√	√
*Pedicularis longiflora*		√
*Polygonum viviparum*		√
*Potentilla anserina*	√	√
*Potentilla multicaulis*		√
*Potentilla saundersiana*	√	√
*Ranunculus brotherusii*	√	√
*Ranunculus membranaceus*	√	
*Saussurea subulisquama*	√	
*Stellera chamaejasme*		√
*Taraxacum maurocarpum*	√	√

## APPENDIX 3

Pollinator species list. √ indicates pollinators existing at Menyuan or Huangyuan plots

**Table:**

Order	Pollinator species	Menyuan	Huangyuan
Diptera	*Bibio* sp1	√	
Diptera	*Bombylius major*	√	√
Diptera	*Cheilosia* sp1		√
Diptera	*Chrysomyia* sp1		√
Diptera	*Conops* sp1	√	
Diptera	*Eristalis tenax*		√
Diptera	*Eurithia* sp1	√	
Diptera	*Eurithia* sp2	√	
Diptera	*Gymnosoma* sp1		√
Diptera	*Lucilia* sp1		√
Diptera	*Lucilia* sp2		√
Diptera	Muscidae sp1	√	
Diptera	Muscidae sp2	√	
Diptera	Muscidae sp3		√
Diptera	*Pales* sp1		√
Diptera	*Pipiza* sp1	√	
Diptera	Sarcophagidae sp1		√
Diptera	*Sphaerophoria* sp1		√
Diptera	*Sphaerophoria viridaenea*		√
Diptera	*Sturmia* sp1	√	
Diptera	Syrphidae sp1		√
Diptera	Syrphidae sp2		√
Diptera	Tachinidae sp1		√
Diptera	Tachinidae sp2	√	√
Hymenoptera	*Andrena* sp1	√	
Hymenoptera	*Anthophora* sp2		√
Hymenoptera	*Apis mellifera*	√	√
Hymenoptera	*Bombus* sp1		√
Hymenoptera	*Bombus asiaticus*	√	√
Hymenoptera	*Bombus ladakhensis*		√
Hymenoptera	*Bombus supremus*	√	√
Hymenoptera	Ichneumonidae sp1		√
Hymenoptera	*Lasioglossum* sp1	√	√
Hymenoptera	*Myrmica* sp1		√
Lepidoptera	*Albulina orbitulus*		√
Lepidoptera	*Celastrina argiola*	√	
Lepidoptera	*Colias fieldii*		√
Lepidoptera	*Colias* sp1	√	
Lepidoptera	*Cupido minimus*	√	
Lepidoptera	*Everes argiades*		√
Lepidoptera	*Satyrium* sp1	√	
Lepidoptera	*Satyrium* sp2	√	
Lepidoptera	*Vanessa cardui*		√

## References

[ece38384-bib-0001] Araújo, M. S. , Bolnick, D. I. , & Layman, C. A. (2011). The ecological causes of individual specialisation. Ecology Letters, 14(9), 948–958. 10.1111/j.1461-0248.2011.01662.x 21790933

[ece38384-bib-0002] Arceo‐Gómez, G. , Abdala‐Roberts, L. , Jankowiak, A. , Kohler, C. , Meindl, G. A. , Navarro‐Fernández, C. M. , Parra‐Tabla, V. , Ashman, T.‐L. , & Alonso, C. (2016). Patterns of among‐ and within‐species variation in heterospecific pollen receipt: the importance of ecological generalization. American Journal of Botany, 103(3), 396–407. 10.3732/ajb.1500155 26507115

[ece38384-bib-0003] Armbruster, W. S. (2017). The specialization continuum in pollination systems: Diversity of concepts and implications for ecology, evolution and conservation. Functional Ecology, 31(1), 88–100. 10.1111/1365-2435.12783

[ece38384-bib-0004] Arroyo‐Correa, B. , Bartomeus, I. , & Jordano, P. (2021). Individual‐based plant–pollinator networks are structured by phenotypic and microsite plant traits. Journal of Ecology, 109(8), 2832–2844. 10.1111/1365-2745.13694

[ece38384-bib-0005] Ballantyne, G. , Baldock, K. C. R. , & Willmer, P. G. (2015). Constructing more informative plant‐pollinator networks: Visitation and pollen deposition networks in a heathland plant community. Proceedings of the Royal Society B‐Biological Sciences, 282(1814), 14–22. 10.1098/rspb.2015.1130 PMC4571695 26336181

[ece38384-bib-0006] Bascompte, J. , & Jordano, P. (2007). Plant‐animal mutualistic networks: The architecture of biodiversity. Annual Review of Ecology, Evolution, and Systematics, 38(1), 567–593. 10.1146/annurev.ecolsys.38.091206.095

[ece38384-bib-0007] Bascompte, J. , Jordano, P. , Melián, C. J. , & Olesen, J. M. (2003). The nested assembly of plant‐animal mutualistic networks. Proceedings of the National Academy of Sciences of the United States of America, 100(16), 9383–9387. 10.1073/pnas.1633576100 12881488 PMC170927

[ece38384-bib-0008] Blüethgen, N. , Menzel, F. , Hovestadt, T. , Fiala, B. , & Blüethgen, N. (2007). Specialization, constraints, and conflicting interests in mutualistic networks. Current Biology, 17(4), 341–346. 10.1016/j.cub.2006.12.039 17275300

[ece38384-bib-0009] Brosi, B. J. (2016). Pollinator specialization: From the individual to the community. New Phytologist, 210(4), 1190–1194. 10.1111/nph.13951 27038018

[ece38384-bib-0010] Brosi, B. J. , & Briggs, H. M. (2013). Single pollinator species losses reduce floral fidelity and plant reproductive function. Proceedings of the National Academy of Sciences of the United States of America, 110(32), 13044–13048. 10.1073/pnas.1307438110 23878216 PMC3740839

[ece38384-bib-0011] CaraDonna, P. J. , Petry, W. K. , Brennan, R. M. , Cunningham, J. L. , Bronstein, J. L. , Waser, N. M. , & Sanders, N. J. (2017). Interaction rewiring and the rapid turnover of plant‐pollinator networks. Ecology Letters, 20(3), 385–394. 10.1111/ele.12740 28156041

[ece38384-bib-0012] Chávez‐González, E. , Vizentin‐Bugoni, J. , Vázquez, D. P. , MacGregor‐Fors, I. , Dáttilo, W. , & Ortiz‐Pulido, R. (2020). Drivers of the structure of plant‐hummingbird interaction networks at multiple temporal scales. Oecologia, 193(4), 913–924. 10.1007/s00442-020-04727-4 32772157

[ece38384-bib-0013] Chittka, L. , Thomson, J. D. , & Waser, N. M. (1999). Flower constancy, insect psychology, and plant evolution. Naturwissenschaften, 86(8), 361–377. 10.1007/s001140050636

[ece38384-bib-0014] Darwin, C. (1876). The effects of cross and self fertilisation in the vegetable kingdom. J. Murray.

[ece38384-bib-0015] de Santiago‐Hernández, M. H. , Martén‐Rodríguez, S. , Lopezaraiza‐Mikel, M. , Oyama, K. , González‐Rodríguez, A. , & Quesada, M. (2019). The role of pollination effectiveness on the attributes of interaction networks: from floral visitation to plant fitness. Ecology, 100(10), e02803. 10.1002/ecy.2803 31240696

[ece38384-bib-0016] Des Roches, S. , Post, D. M. , Turley, N. E. , Bailey, J. K. , Hendry, A. P. , Kinnison, M. T. , Schweitzer, J. A. , & Palkovacs, E. P. (2018). The ecological importance of intraspecific variation. Nature Ecology & Evolution, 2(1), 57–64. 10.1038/s41559-017-0402-5 29203921

[ece38384-bib-0017] Dupont, Y. L. , Trøjelsgaard, K. , Hagen, M. , Henriksen, M. V. , Olesen, J. M. , Pedersen, N. M. E. , & Kissling, W. D. (2014). Spatial structure of an individual‐based plant‐pollinator network. Oikos, 123(11), 1301–1310. 10.1111/oik.01426

[ece38384-bib-0018] Dupont, Y. L. , Trøjelsgaard, K. , & Olesen, J. M. (2011). Scaling down from species to individuals: A flower‐visitation network between individual honeybees and thistle plants. Oikos, 120(2), 170–177. 10.1111/j.1600-0706.2010.18699.x

[ece38384-bib-0019] Fang, Q. , & Huang, S. Q. (2013). A directed network analysis of heterospecific pollen transfer in a biodiverse community. Ecology, 94(5), 1176–1185. 10.1890/12-1634.1 23858657

[ece38384-bib-0020] Fang, Q. , & Huang, S.‐Q. (2016). A paradoxical mismatch between interspecific pollinator moves and heterospecific pollen receipt in a natural community. Ecology, 97(8), 1970–1978. 10.1002/ecy.1433 27859194

[ece38384-bib-0021] Freitas, L. (2013). Concepts of pollinator performance: Is a simple approach necessary to achieve a standardized terminology? Brazilian Journal of Botany, 36(1), 3–8. 10.1007/s40415-013-0005-6

[ece38384-bib-0022] Guimarães, P. R. (2020). The structure of ecological networks across levels of organization. Annual Review of Ecology, Evolution, and Systematics, 51(1), 433–460. 10.1146/annurev-ecolsys-012220-120819

[ece38384-bib-0023] Ings, T. C. , Montoya, J. M. , Bascompte, J. , Blüthgen, N. , Brown, L. , Dormann, C. F. , & Woodward, G. (2009). Ecological networks—Beyond food webs. Journal of Animal Ecology, 78(1), 253–269. 10.1111/j.1365-2656.2008.01460.x 19120606

[ece38384-bib-0024] Johnson, S. D. , & Steiner, K. E. (2000). Generalization versus specialization in plant pollination systems. Trends in Ecology & Evolution, 15(4), 140–143. 10.1016/s0169-5347(99)01811-x 10717682

[ece38384-bib-0025] Kaiser‐Bunbury, C. N. , Mougal, J. , Whittington, A. E. , Valentin, T. , Gabriel, R. , Olesen, J. M. , & Blüthgen, N. (2017). Ecosystem restoration strengthens pollination network resilience and function. Nature, 542(7640), 223–227. 10.1038/nature21071 28135718

[ece38384-bib-0026] Lázaro, A. , Hegland, S. J. , & Totland, Ø. (2008). The relationships between floral traits and specificity of pollination systems in three Scandinavian plant communities. Oecologia, 157(2), 249–257. 10.1007/s00442-008-1066-2 18506487

[ece38384-bib-0027] Lucas, A. , Bodger, O. , Brosi, B. J. , Ford, C. R. , Forman, D. W. , Greig, C. , Hegarty, M. , Neyland, P. J. , & de Vere, N. (2018). Generalisation and specialisation in hoverfly (Syrphidae) grassland pollen transport networks revealed by DNA metabarcoding. Journal of Animal Ecology, 87(4), 1008–1021. 10.1111/1365-2656.12828 PMC6032873 29658115

[ece38384-bib-0028] Memmott, J. (1999). The structure of a plant‐pollinator food web. Ecology Letters, 2(5), 276–280. 10.1046/j.1461-0248.1999.00087.x 33810635

[ece38384-bib-0029] Morales, C. L. , & Traveset, A. (2008). Interspecific pollen transfer: Magnitude, prevalence and consequences for plant fitness. Critical Reviews in Plant Sciences, 27(4), 221–238. 10.1080/07352680802205631

[ece38384-bib-0030] Olesen, J. M. , Bascompte, J. , Dupont, Y. L. , Elberling, H. , Rasmussen, C. , & Jordano, P. (2011). Missing and forbidden links in mutualistic networks. Proceedings of the Royal Society B: Biological Sciences, 278(1706), 725–732. 10.1098/rspb.2010.1371 PMC3030842 20843845

[ece38384-bib-0031] Olesen, J. M. , Bascompte, J. , Dupont, Y. L. , & Jordano, P. (2007). The modularity of pollination networks. Proceedings of the National Academy of Sciences of the United States of America, 104(50), 19891–19896. 10.1073/pnas.0706375104 18056808 PMC2148393

[ece38384-bib-0032] Olesen, J. M. , Bascompte, J. , Elberling, H. , & Jordano, P. (2008). Temporal dynamics in a pollination network. Ecology, 89(6), 1573–1582. 10.1890/07-0451.1 18589522

[ece38384-bib-0033] Olesen, J. M. , Dupont, Y. L. , O'Gorman, E. J. , Ings, T. C. , Layer, K. , Melián, C. J. , & Woodward, G. (2010). From Broadstone to Zackenberg: Space, time and hierarchies in ecological networks. Advances in Ecological Research, 42, 1–69. 10.1016/s0065-2504(10)42001-2

[ece38384-bib-0034] Petanidou, T. , Kallimanis, A. S. , Tzanopoulos, J. , Sgardelis, S. P. , & Pantis, J. D. (2008). Long‐term observation of a pollination network: Fluctuation in species and interactions, relative invariance of network structure and implications for estimates of specialization. Ecology Letters, 11(6), 564–575. 10.1111/j.1461-0248.2008.01170.x 18363716

[ece38384-bib-0035] R Development Core Team (2019). R: A language and environment for statistical computing. R Core Team.

[ece38384-bib-0036] Schwarz, B. , Vázquez, D. P. , CaraDonna, P. J. , Knight, T. M. , Benadi, G. , Dormann, C. F. , Gauzens, B. , Motivans, E. , Resasco, J. , Blüthgen, N. , Burkle, L. A. , Fang, Q. , Kaiser‐Bunbury, C. N. , Alarcón, R. , Bain, J. A. , Chacoff, N. P. , Huang, S.‐Q. , LeBuhn, G. , MacLeod, M. , … Fründ, J. (2020). Temporal scale‐dependence of plant–pollinator networks. Oikos, 129(9), 1289–1302. 10.1111/oik.07303

[ece38384-bib-0037] Seifert, C. L. , Jorge, L. R. , Volf, M. , Wagner, D. L. , Lamarre, G. P. A. , Miller, S. E. , Gonzalez‐Akre, E. , Anderson‐Teixeira, K. J. , & Novotný, V. (2021). Seasonality affects specialisation of a temperate forest herbivore community. Oikos, 130(9), 1450–1461. 10.1111/oik.08265

[ece38384-bib-0038] Souza, C. S. , Maruyama, P. K. , Aoki, C. , Sigrist, M. R. , Raizer, J. , Gross, C. L. , & de Araujo, A. C. (2018). Temporal variation in plant‐pollinator networks from seasonal tropical environments: Higher specialization when resources are scarce. Journal of Ecology, 106(6), 2409–2420. 10.1111/1365-2745.12978

[ece38384-bib-0039] Stebbins, G. L. (1970). Adaptive radiation of reproductive characteristics in angiosperms, I: Pollination mechanisms. Annual Review of Ecology and Systematics, 1(1), 307–326. 10.1146/annurev.es.01.110170.001515

[ece38384-bib-0040] Tonos, J. , Razafindratsima, O. H. , Fenosoa, Z. S. E. , & Dunham, A. E. (2021). Individual‐based networks reveal the highly skewed interactions of a frugivore mutualist with individual plants in a diverse community. Oikos. 10.1111/oik.08539

[ece38384-bib-0041] Traveset, A. , Olesen, J. M. , Nogales, M. , Vargas, P. , Jaramillo, P. , Antolín, E. , & Heleno, R. (2015). Bird‐flower visitation networks in the Galapagos unveil a widespread interaction release. Nature Communications, 6, 6376. 10.1038/ncomms7376 25757227

[ece38384-bib-0042] Tur, C. , Vigalondo, B. , Trøjelsgaard, K. , Olesen, J. M. , & Traveset, A. (2014). Downscaling pollen‐transport networks to the level of individuals. Journal of Animal Ecology, 83(1), 306–317. 10.1111/1365-2656.12130 24107193

[ece38384-bib-0043] Vitt, P. , Havens, K. , Jolls, C. L. , & Knight T. M. (2020). Temporal variation in the roles of exotic and native plant species in plant‐pollinator networks. Ecosphere, 11(2). e02981. 10.1002/ecs2.2981

[ece38384-bib-0044] Wang, H. , Cao, G. X. , Wang, L. L. , Yang, Y. P. , Zhang, Z. Q. , & Duan, Y. W. (2017). Evaluation of pollinator effectiveness based on pollen deposition and seed production in a gynodioecious alpine plant, *Cyananthus delavayi* . Ecology and Evolution, 7(20), 8156–8160. 10.1002/ece3.3391 29075439 PMC5648671

[ece38384-bib-0045] Wang, L. L. , Yang, N. C. , Chen, M. Y. , Yang, Y. P. , & Duan, Y. W. (2021). Polyploidization and sexual dimorphism of floral traits in a subdioecious population of *Dasiphora glabra* . Journal of Plant Ecology, 14(2), 229–240. 10.1093/jpe/rtaa089

[ece38384-bib-0046] Waser, N. M. (1978). Interspecific pollen transfer and competition between co‐occurring plant species. Oecologia, 36(2), 223–236. 10.1007/bf00349811 28309130

[ece38384-bib-0047] Waser, N. M. (1986). Flower constancy: Definition, cause, and measurement. American Naturalist, 127(5), 593–603. 10.1086/284507

[ece38384-bib-0048] Waser, N. M. , Chittka, L. , Price, M. V. , Williams, N. M. , & Ollerton, J. (1996). Generalization in pollination systems, and why it matters. Ecology, 77(4), 1043–1060. 10.2307/2265575

